# Strategies to increase childhood tuberculosis case detection at the primary health care level: Lessons from an active case finding study in Zambia

**DOI:** 10.1371/journal.pone.0288643

**Published:** 2023-07-19

**Authors:** Mary Kagujje, Sarah Nyangu, Minyoi M. Maimbolwa, Brian Shuma, Lilungwe Mutti, Paul Somwe, Nsala Sanjase, Chalilwe Chungu, Andrew D. Kerkhoff, Monde Muyoyeta

**Affiliations:** 1 Tuberculosis Department, Centre of Infectious Disease Research in Zambia, Lusaka, Zambia; 2 Strategic Information Department, Centre of Infectious Disease Research in Zambia, Lusaka, Zambia; 3 Programs, Catholic Relief Services, Lusaka, Zambia; 4 Department of Medicine, University of San Francisco California, San Francisco, California, United States of America; Shandong Public Health Clinical Center: Shandong Provincial Chest Hospital, CHINA

## Abstract

**Introduction:**

In high TB burden settings, it is estimated that 10–20% of total notifications should be children, however, currently only 6–8% of the total TB notifications in Zambia are children. We assessed whether the implementation of a multicomponent strategy, at primary healthcare facilities, that systematically targets barriers at each step of the childhood TB diagnostic cascade can increase childhood TB case detection.

**Methods:**

We conducted a controlled, interrupted time series analysis to compare childhood TB case notifications before (January 2018—December 2019), and during implementation (January 2020—September 2021) in two intervention and two control Level 1 hospitals in Lusaka, Zambia. At each of the intervention facilities, we implemented a multicomponent strategy constituting: (1) capacity development on childhood TB and interpretation of chest x-ray, (2) TB awareness-raising and demand creation activities, (3) setting up fast track TB services, (4) strengthening of household contact tracing, and (5) improving access to digital chest X-ray for TB screening and Xpert MTB/Rif Ultra for TB diagnosis, through strengthening sample collection in children.

**Findings:**

Among 5,150 children < 15 years screened at the two intervention facilities during the study period, 503 (9.8% yield) were diagnosed with TB. Of these, 433 (86.1%) were identified through facility-based activities (10.5% yield) and 70 (13.9%) were identified through household contact tracing (6.9% yield). Overall, 446 children (88.7%) children with TB were clinically diagnosed. Following implementation of the multicomponent strategy, the proportion children contributed to total TB notifications immediately changed by +1.5% (95%CI: -3.5, 6.6) and -4.4% (95%CI: -7.5, 1.4) at the intervention and control sites, respectively (difference 6.0% [95%CI: -0.7, 12.7]), p = 0.08); the proportion of childhood notifications increased 0.9% (95%CI: -0.7, 2.5%) each quarter at the intervention sites relative to pre-implementation trends, while declining 1.2% (-95%CI: -1.8, -0.6) at the control sites (difference 2.1% [95%CI: 0.1, 4.2] per quarter between, p = 0.046); this translated into 352 additional and 85 fewer childhood TB notifications at the intervention and control sites, respectively, compared to the pre-implementation period.

**Conclusion:**

A standardized package of strategies to improve childhood TB detection at primary healthcare facilities was feasible to implement and was associated with a sustained improvement in childhood TB notifications.

## Introduction

Of the 10 million estimated tuberculosis (TB) cases globally in 2020, 11% (1.1 million) were among children aged less than 15 years [1]. Of these estimated children with TB, 63% didn’t access TB diagnosis and treatment or were not reported to the national TB programs [1]. Modelling estimates indicate that 10–20% of the total TB burden in high TB burden countries is among children under the age of 15 years [2]. In Zambia, a high TB burden country with an estimated TB incidence of 319 per 100,000 population [1], only 6% of its notified 40,000 TB patients in 2021 were among children [1, 3]. Further, the country failed to diagnose, treat, and notify 50% of its estimated childhood TB cases [1, 3]. Delay and failure to diagnose TB in children leads to increased risk of long-term sequelae, disability, and death from a disease that is entirely curable [4–6].

Commonly, children with signs and symptoms of TB first present to primary healthcare facilities as TB symptomology in children are similar to those of other common illnesses [7–10]. Presentation to these facilities provides an opportunity for early diagnosis of TB in children. However, due to the complexities of diagnosing childhood TB, children are often referred to higher-level referral hospitals and specialized pediatric units, resulting in delayed diagnosis [7, 8].

Delay and failure to diagnose childhood TB is due to various factors that include the nonspecific nature of TB presentation (i.e., signs and symptoms) in children, challenges in expectorating sputum which then necessitates invasive procedures to collect sputum or other extra-pulmonary specimens, low bacillary load making a microbiological confirmation of TB difficult, as well as less specific radiological changes on chest X-ray (CXR) compared to adults with TB [11–15]. These factors are compounded by health system and social factors which include limited access to sensitive TB diagnostic tools, low index of suspicion of childhood TB among healthcare workers, and limited competence, especially among providers at primary healthcare facilities, to make a clinical diagnosis of TB. The fact that children are dependent on their parents or caregivers to be brought to the hospital may also result in further delay [15–18].

We conducted a quasi-experimental study using controlled interrupted time series analyses to evaluate whether the implementation of a multicomponent strategy that systematically target barriers at each step of the childhood TB diagnostic cascade would result in an increase in childhood TB case detection at primary health facilities. Herein we document our findings and share lessons learned during the study.

## Methods

### Study design and setting

A quasi-experimental study using a controlled, interrupted time series analysis, assessed all childhood (<15 years of age) TB case notifications before (January 2018 to December 2019) and during (January 2020 –September 2021) implementation of a multicomponent strategy to bolster childhood TB diagnosis at two intervention and two control sites.

**Fig 1:**
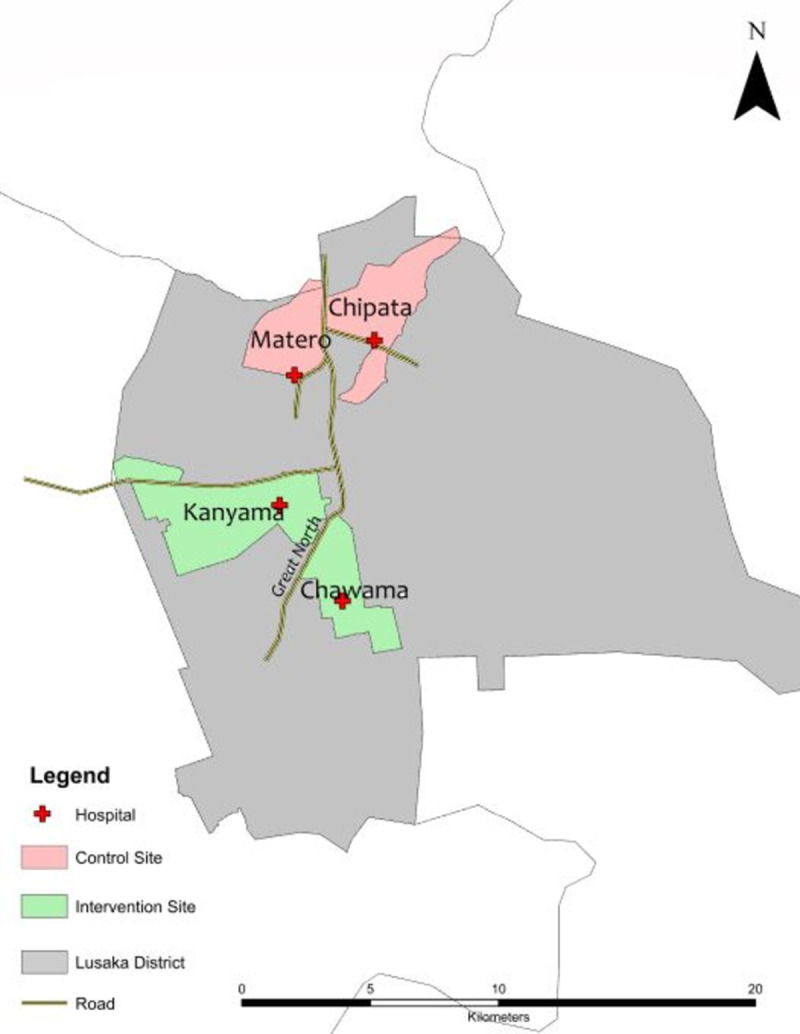
Geographical locations of intervention and control sites under the study.

Two intervention sites, Kanyama and Chawama hospitals (Fig 1), were purposefully selected because they each have high TB notifications (>1000 TB notifications per year), yet they had a low proportion of childhood TB notifications (∼5%). The control sites, Matero and Chipata hospitals, were selected because they were comparable to the intervention sites both in terms of socio-economic status of the catchment population and having a high number of annual TB notification rate (718 vs. 694 per 100,000 population, for the control and intervention sites respectively in 2019) with a very low proportion being among children (6% vs. 4%, respectively in 2019). Additionally, the paediatric catchment population (0–14.9 years) of the two control sites (352,114) and two intervention sites (302,975) were also similar (facility catchment population data, unpublished). All intervention and control sites are public hospitals situated in Lusaka urban district within 5-10km from the central business district. Each facility has similar TB diagnostic infrastructure and utilize the same standardized, national protocols for TB case screening and diagnosis. All the sites were first-level hospitals at the time of the study, but they have since been upgraded to second-level hospitals.

### Study procedures and description of childhood TB detection strategy components

Prospective study activities were conducted at the two intervention sites between January 2020 and September 2021. At each intervention site, we implemented and sustained a multicomponent strategy that included: (1) capacity development on childhood TB among healthcare workers, (2) TB awareness raising and demand creation, (3) setting up fast track TB evaluation services, (4) strengthening of household contact tracing, and (5) improving access to sensitive screening tools and diagnostic tests for TB, including digital CXR, and bacteriological examination of paediatric clinical specimens for TB using Xpert MTB/RIF Ultra(Cepheid, Sunnyvale California). The discrete strategy components sought to address hypothesized key barriers at each step of the pediatric TB diagnostic cascade and were informed by local programmatic data, as well as insights from local stakeholders and a review of the literature from comparable settings. Each component of the strategy is described in greater detail below.

#### Component #1: Capacity development on childhood TB

Facility nurses and clinicians (clinical officers and medical doctors) underwent a one-week training using the national training package that covered (a) identification of children with presumptive TB, (b) the use of the national childhood TB diagnostic algorithm, and (c) collection of respiratory and non-respiratory samples in children (gastric and nasopharyngeal aspirates), among other topics. Additionally, clinicians participated in a four-week online training on radiological diagnosis of TB which included interpretation of chest x-rays of children.

Community health care workers underwent a 3-day refresher training on TB, which included dedicated lectures on household contact tracing and identification of children with presumptive TB.

#### Component #2: Awareness and demand creation activities

The study provided a sensitization guide with standardized messages on childhood TB to the community health workers at each intervention site. A dedicated community health worker assigned to each department used this guide to provide health education to parents/caregivers seeking services for their children in different departments of the health facility–this was carried out every two hours each day. After sensitization, parents/caregivers were invited to bring their children to the fast-track TB screening services.

#### Component #3: Fast-track TB services

A desk was set up at the chest clinic for each intervention site to provide fast-track TB services to children and was staffed by a clinician and a community health worker. Children with possible TB symptoms (cough, poor weight gain or weight loss, fever, night sweats, reduced playfulness, poor appetite, lethargy, and failure to thrive) [19] were referred to the fast track desk by health care workers and community health workers from all site departments, especially the pediatric anti-retroviral therapy (ART) clinic, nutritional department, and outpatient department (OPD) for further TB evaluation. Additionally, the fast-track desk received self-referrals from parents/caregivers who wanted their children evaluated for TB.

#### Component #4: Strengthening household contact tracing

Community health workers were used to conduct contact tracing of bacteriologically confirmed adult TB patients and reverse contact tracing of children diagnosed with TB. During home visits, they provided health education to household members of the index patient on various aspects of TB after which they invited all contacts (regardless of symptoms) for TB screening at the health facility by a health care worker. When a contact was unwilling to visit the health facility, symptom screening was done by a community health worker and if the contact was symptomatic for TB, and where feasible, a spontaneously expectorated sputum sample was collected and sent to the health facility for TB testing using Xpert MTB/RIF Ultra. During periods of high COVID-19 transmission, the study team conducted symptom-based screening of household contacts via phone calls and invited only the symptomatic contacts for further evaluation at the health facility.

#### Component #5: Improving access to sensitive TB screening and diagnostic tests

The study provided an ultra-portable digital Fujifilm X-ray machine (Fujifilm, Japan), which was shared by the two intervention sites during the study. All children <15 years of age at the intervention sites, regardless of symptoms status, identified at the health facility through fast-track TB services or through household contact tracing, were screened for pulmonary TB using CXR when the X-ray unit was available. All CXR images were interpreted same-day by the requesting clinicians to identify any abnormalities. Symptom screening and CXR screening were in used in parallel to improve the sensitivity of the screening algorithm and maximise case detection in accordance with WHO recommendations [20]. Children living with HIV and under 5 contacts to TB cases with normal CXR and without TB symptoms were offered TB Preventive Treatment (TPT)– 6 months of isoniazid–in accordance with national guidelines [21] and linked to the local chest clinic for adherence support and treatment monitoring. Children meeting any of the following criteria were considered presumptive TB patients: abnormal CXR, signs and symptoms suggestive of TB or being a contact to a TB patient.

Sputum or gastric aspirates were collected from all presumptive TB patients and these samples were subjected to microbiological testing for TB using Xpert MTB/RIF Ultra. The TB Lipoarabinomannan (LAM) rapid urine assay (Abott, Palantine, Illinois) was utilized if presumptive TB patients were either (a) HIV positive (regardless of CD4 count or WHO clinical staging), in line with the WHO recommendations on the use of LAM among symptomatic individuals [22], or (b) if the patient was <5 years of age irrespective of HIV status, based on evidence suggesting the incremental value of LAM testing in HIV negative children <5 years old [23, 24]. All respiratory and urine samples were processed and examined by the clinical laboratories based at the intervention sites in accordance with national standard operating procedures for Xpert MTB/RIF Ultra and urine LAM, respectively

All children with MTB detected on Xpert MTB/RIF Ultra (including trace positive results) were considered positive, while clinical discretion (e.g., in the context of clinical history and CXR results) was used for the interpretation of Alere LAM results due to associated high rates of false positivity [25], and the use of the tool outside the WHO recommendations [22]. Among children without a microbiological confirmation of TB, a clinical diagnosis of TB was made in accordance with international recommendations, if they had any current symptom of TB and met any two of the four following criteria: (1) close contact with a bacteriologically confirmed TB patient, (2) abnormal respiratory sign or signs of extrapulmonary TB, (3) abnormal CXR, or (4) abnormal supportive investigations for TB(e.g., ultrasound scan, an x-ray of the joints, echocardiogram, etc.) [26]. The facility clinicians consulted the study clinicians when they were not sure about the diagnosis of TB in a child they were evaluating. A decision on the final diagnosis and whether to start treatment was determined through consensus.

#### Description of standard of care services at control sites

During the study period, the two control sites provided standard of care services for childhood TB diagnosis and treatment as defined by the Zambian Ministry of Health [19]–no new interventions or strategies were implemented. Childhood TB case finding was mainly passive (e.g., testing only among symptomatic children being brought in for evaluation) through the outpatient department, and not all presumptive children had access to CXR. Childhood TB screening algorithms were available in the control sites (in accordance with national guidelines); however, they may not have been routinely used by healthcare providers to guide diagnosis of childhood TB. Of note, during the study period (from July 2020 onward), the National TB and Leprosy Programme (NTLP) started implementing a multi-component strategy, including strengthening facility-based case finding and contact tracing, across the country, to mitigate the impact of COVID on TB case finding [27]. However, this strategy predominantly focused on bolstering adult TB notifications.

### Data sources

Two data sources were used for the analysis: facility-specific electronic study screening registers and the district quarterly TB notification reports. The study register at each facility collected data on participant demographics and clinical parameters, entry point (whether through facility-based activities or household contact tracing) and the final outcome of TB evaluation (e.g., TB diagnosed or not diagnosed). The quarterly district notification reports provided information on TB patients initiated on treatment that were notified by each facility to the district, stratified by age and health facility.

### Data analysis

The primary outcome of this study was to determine the yield of children diagnosed with TB with reference to the total notifications of each site. Secondary outcomes included determining the number of children identified as presumptive TB patients both through facility-based activities and contact tracing, the proportion of children with a CXR undertaken, the proportion of children examined using Xpert MTB/RIF Ultra, and the yield of TB among children identified through the facility-based activities versus from contact tracing and the additionality of the interventions.

All data analysis was conducted using STATA version 17.0 (StataCorp, College Station, Texas, USA). The characteristics of participants and the TB diagnostic yield under the facility-based interventions and household contact tracing interventions were compared using chi-squared tests and student t-tests as appropriate. To evaluate the effect of the multicomponent strategy to improve childhood TB detection, we undertook a before and after comparison and a controlled, interrupted time series analysis using an Ordinary Least Squares (OLS) segmented linear regression model using the user-written "ITSA" command [28]. In the before and after comparison, we compared the same time periods (pre-intervention period of January 2018—September 2019 and the intervention period of January 2020—September 2021) to account for seasonal variation in TB notifications. In the interrupted time series analysis, we assessed (a) quarterly childhood TB notifications and (b) the proportional contribution of childhood TB notifications among all TB notifications each quarter at the two intervention and two control sites, before and during implementation of the strategy; these were assessed overall and by age group (< 5 years, 5–14.9 years). The pre-implementation period was January 2018 to December 2019 while the intervention period was January 2020 to September 2021. Cumby-Huizinga test was used to check for the presence of autocorrelation, while Newey-West standard errors were used to account for autocorrelation.

### Ethical considerations

Ethical approval was obtained from the University of Zambia Biomedical research ethics committee (Reference Number: 635–2020). The study obtained a waiver of written informed consent from the research ethics committee.

## Results

### Characteristics of children screened at intervention sites

**Fig 2:**
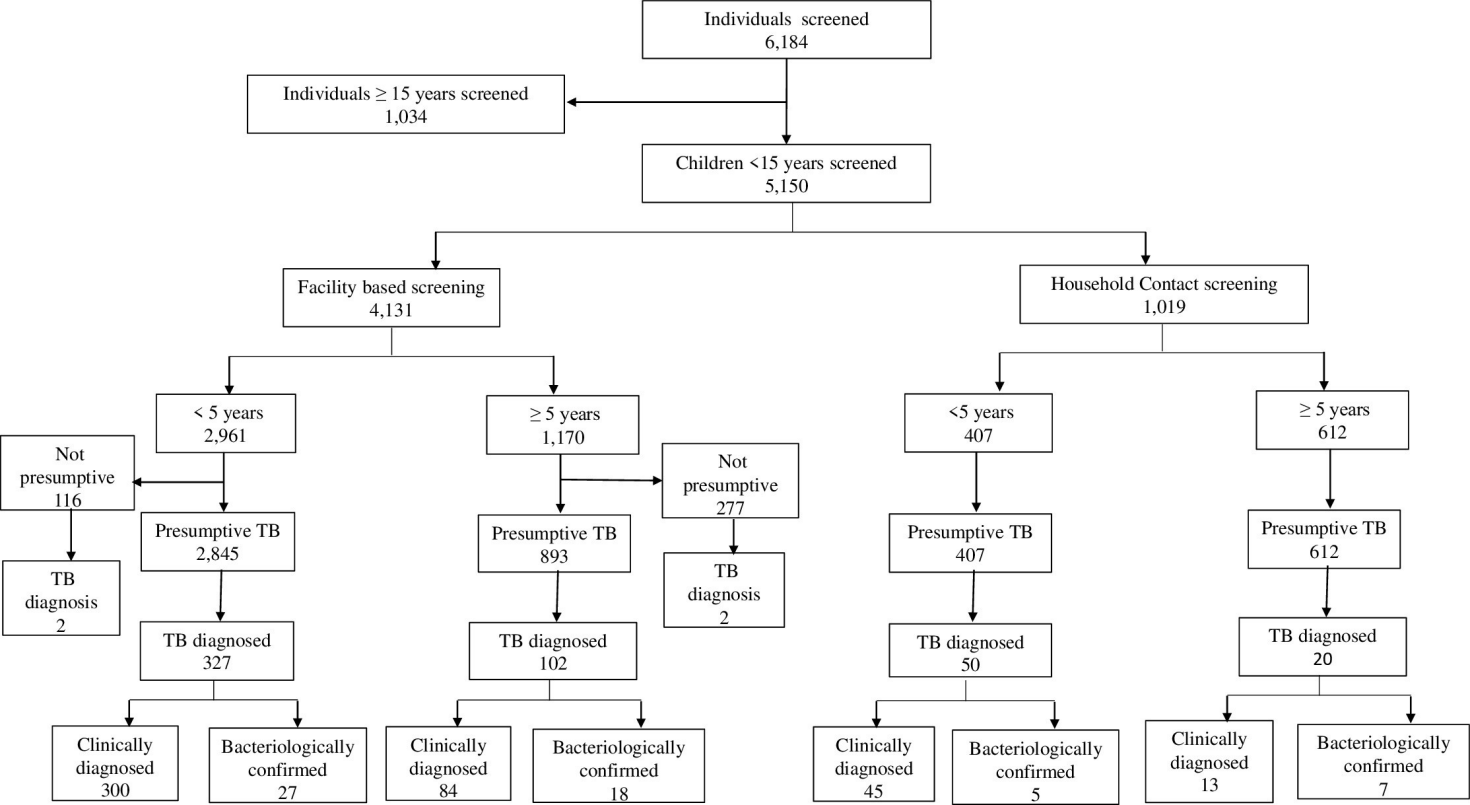
Study flow diagram.

Overall, 5,150 children <15 years were screened for TB at the two intervention sites during the study period, of which 4,131 (80.2%) were identified through facility-based activities and 1,019 (19.8%) were through household contact tracing (**Fig 2**).

Children screened through the facility-based activities were more likely to be younger, HIV positive, and have more symptoms suggestive of TB (**Table 1**); they were also more likely to undergo CXR and have an abnormal CXR result. There were no differences in the two groups with respect to gender and a history of previous TB.

**Table 1:** Characteristics of children screened through facility-based activities and contact tracing activities.

	Overall (n = 5,150)	Facility-based activities (n = 4,131)	Household contact tracing (n = 1,019)	P-value
**Male sex**	2,630 (51.2)	2,128 (51.5)	502(49.3)	0.191
**Mean Age (sd)**	3.4 (4.0)	3.3 (4.0)	6.3 (4.2)	<0.001
<5 years	3,368 (65.4)	2,961 (71.7)	407 (39.9)	<0.001
5–14.9 years	1,782 (34.6)	1,170 (28.3)	612 (60.1)	
**HIV positive status**	554 (11.0)	546 (13.2)	8 (0.8)	<0.001
**Previous TB**	29 (0.6)	127 (0.6)	2 (0.2)	0.08
**Symptoms** ^**#**^				
No symptoms	1,140 (22.1)	496 (12.1)	644 (63.2)	<0.001
1 Symptom	969 (18.8)	789 (19.1)	180 (17.7)	
2 or more symptoms	3,041 (59.1)	2,846 (68.9)	195 (19.1)	
**Had CXR undertaken**	2,579 (50.1)	2,148 (52.0)	431 (42.3)	<0.001
Abnormal CXR*	1,207 (46.8)	1,047 (48.7)	160 (37.1)	<0.001
**TB Diagnosed**	503 (9.8)	433 (10.5)	70 (6.9)	<0.001
Bacteriologically confirmed	57 (11.3)	45 (10.4)	12 (17.1)	0.10
Clinically diagnosed	446 (88.7)	388 (89.6)	58 (82.7)	

*2571 did not have CXR done or CXR read

^#^Symptoms assessed included cough, night sweats, fevers, or unintentional weight loss

All values represent n (%) except where explicitly noted.

Abbreviations: HHC- Household contact tracing, TB- tuberculosis, CXR- chest x-ray

### TB diagnoses made among children at intervention sites

Overall, 503 (yield 9.8% [95%CI: 9.0–10.6%]) children were diagnosed with TB at the two intervention sites during the study period. Children identified through facility-based activities (n = 4,131) were more likely than children identified from household contact tracing (n = 1,019) to have TB diagnosed (yield 10.5% [95%CI: 9.6–11.5] vs. 6.9% [95%CI: 5.4–8.6], p<0.001).

Among children diagnosed with TB screened through facility-based activities (n = 433), 329 (76.0% of facility-based TB diagnoses; yield 11.1%) and 104 (24.0% of facility-based TB diagnoses; yield 8.9%) were among children <5 and ≥ 5 years old, respectively (p = 0.036 for difference in yield). The proportion of children with TB identified through facility-based activities that had a bacteriologically confirmed diagnosis was 10.4% (n = 45) overall and was 8.2% (n = 27) and 17.7% (n = 17) among <5 years and ≥5 years, respectively.

Of children identified with TB through household contact tracing (n = 70), 50 (71.4% of household contact tracing TB diagnoses; yield 12.3%) were among those <5 years old, while 20 (28.6% of household contact tracing TB diagnoses; yield 3.3%) were among those ≥ 5 years old (p<0.001 for difference in yield). The proportion of children with TB identified through household contact tracing that had a bacteriologically confirmed diagnosis was 17.1% (n = 12) overall and was 10.0% (n = 5) and 35.0% (n = 7) among <5 years and ≥ 5 years, respectively.

### Impact on TB notifications before and during implementation

Next, we used district-level TB notifications reports to evaluate the effect of implementing the multicomponent strategy to increase childhood TB detection. Of note, the district monthly TB notification reports had a slightly higher number of childhood TB notifications in the intervention sites compared to the number of childhood TB diagnoses in the study register (508 compared to 503) (**S1 Table**).

At the outset of the pre-implementation period, intervention and control sites had a similar number of childhood TB notifications per quarter (16.2 vs. 21.8, p = 0.16); quarterly childhood TB notifications were significantly increasing in both intervention (+1.8, p = 0.001) and control (+6.0, p = 0.001) sites prior to implementation, although the trend was higher in control sites (difference -4.2, p<0.001) (**Table 2)**. Immediately following implementation, childhood notifications were relatively unchanged (+12.1, p = 0.64) in intervention sites while decreasing by -37.2 (p = 0.001) in intervention sites (difference +49.3, p = 0.07). Compared to pre-implementation trends, quarterly childhood TB notifications were similar at intervention sites (+8.3, p = 0.33), while they continued to decline at control sites (-7.8, p = 0.001) (difference +16.1, p = 0.06) (**Table 2)**.

**Table 2:** Childhood TB notifications before and during implementation of strategy to improve TB detection among children by intervention and control sites.

	TB Notifications Before strategy implementation (Jan. 2018 –Dec. 2019) [Period #1]			TB notifications During strategy implementation (Jan. 2020 –Sept. 2021) [Period #2]		
	Initial number of quarterly notifications (95%CI)	Quarterly trend (95%CI)		Immediate impact on quarterly notifications	Quarterly notification trend	
				Number (95%CI)	Relative to period #1 (95%CI)	Overall during period #2 (95%CI)
**All children**						
Intervention sites	16.2 (13.5, 18.8)	+1.8^**^ (0.9, 2.6)		+12.1 (-42.9, 67.1)	+8.3 (-9.6, 26.2)	+10.0 (-7.5, 27.6)
Control sites	21.8 (9.7, 33.9)	+6.0^**^ (3.2, 8.7)		-37.2** (-54.1, -20.2)	-7.8^**^ (-11.8, -3.8)	-1.8 (-4.5, 0.9)
*Difference (relative to control sites)*	-5.7 (-13.8, 2.5)	-4.2^***^ (-6.2, -2.2)		+49.3^#^ (-4.3, 102.8)	+16.1^#^ (-1.0, 33.1)	+11.9^#^ (-4.8, 28.5)
**Age <5 years old**						
Intervention sites	7.6 (5.6, 9.6)	+0.3 (-0.2, 0.8)		+21.9 (-37.5, 81.2)	+6.7 (-11.2, 24.6)	+7.0 (-10.8, 24.8)
Control sites	13.2 (4.0, 22.3)	+3.8^**^ (1.8, 5.8)		-24.0^**^ (-38.2, -9.9)	-5.0^**^ (-8.0, -2.0)	-1.3 (-3.3, 0.8)
*Difference*	-5.6 (-12.2, 1.0)	-3.5^***^ (-5.1, -1.9)		+45.9^#^ (-6.2, 98.0)	+11.7 (-4.7, 28.1)	+8.2 (-8.0, 24.5)
**Age 5 to 14.9 years old**						
Intervention sites	8.6 (7.3, 9.9)	+1.5^***^ (1.1, 2.0)		-9.8^**^ (-14.8, -4.7)	+1.6^*^ (0.2, 2.9)	+3.1^***^ (1.8, 4.3)
Control sites	8.7 (3.3, 14.1)	+2.2^**^ (0.8, 3.5)		-13.1^**^ (-20.1, -6.2)	-2.8^**^ (-4.4, -1.1)	-0.6 (-1.4, 0.2)
*Difference*	-0.1 (-5.6, 5.4)	-0.7 (-2.2, 0.8)		+3.4 (-5.7, 12.5)	+4.3^**^ (1.4, 7.2)	+3.6^**^ (1.4, 5.8)

^#^p = 0.05–0.10

*p<0.05

**p<0.01

***p<0.001

Overall, this translated to 352 additional (225.6% higher) and 85 fewer (31% lower) childhood notifications in the implementation period compared to the pre-implementation period at intervention and control sites, respectively (**S1 Table)**. When evaluating effects by age group, implementation notifications increased among those <5 and 5–14.9 years old relative pre-implementation, although trends only reached significance in 5–14.9-year-olds; notably, notifications significantly declined among both <5 and 5–14.9 years olds compared to pre-implementation trends in the control sites (**Table 2)**.

**Fig 3:**
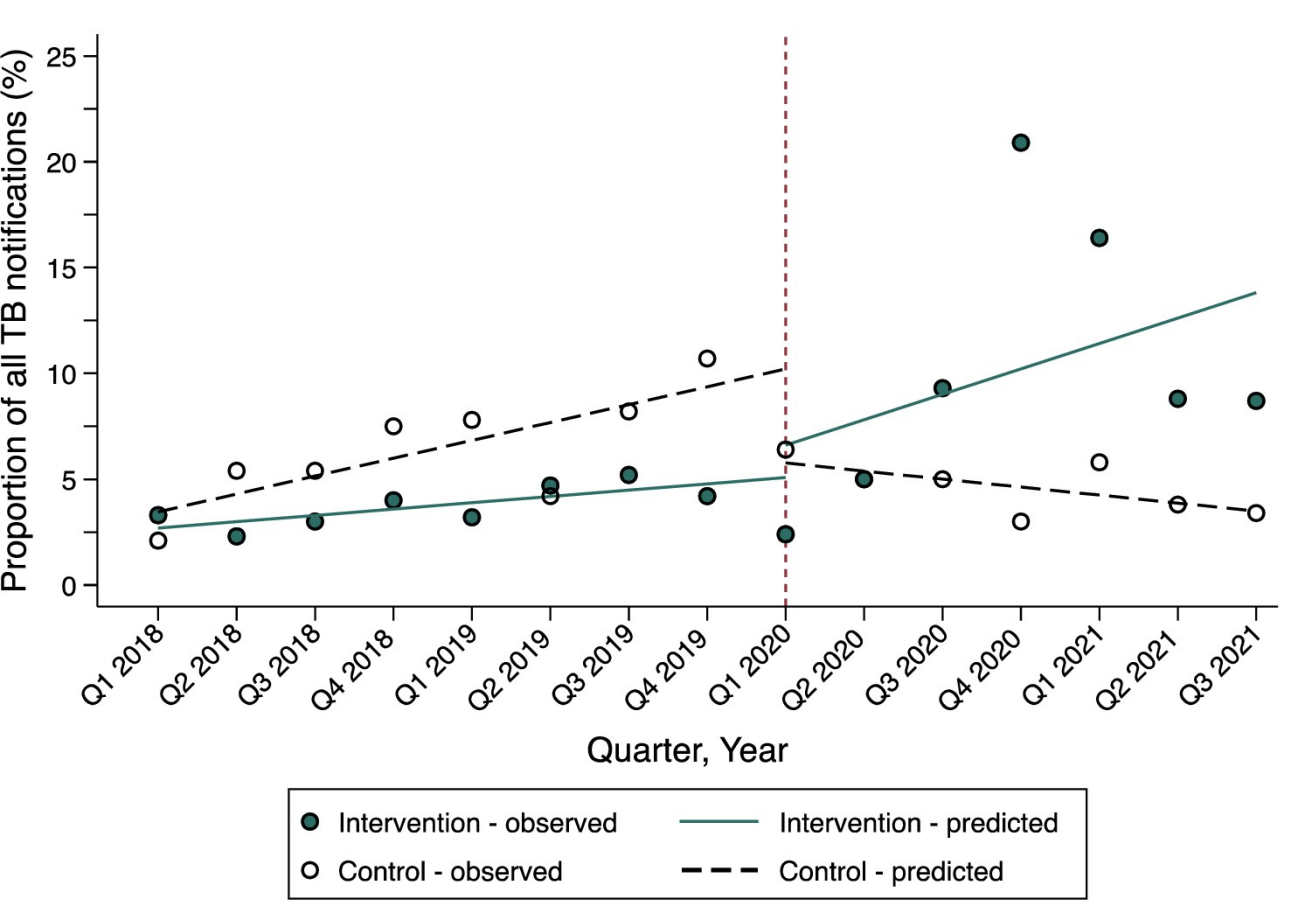
The proportion of childhood TB notifications among all TB notifications at intervention and controls sites, before and during implementation of a multicomponent strategy to improve childhood TB detection.

Finally, we assessed changes in the proportional contribution of childhood TB notifications to all site notifications before and during implementation (**Fig 3 and S2 Table).** During the initial pre-implementation period, childhood notifications accounted for 2.7% and 3.5% of total notifications at intervention and control sites, respectively (p = 0.13); this increased by 0.3% (p<0.001) per quarter in the pre-implementation period at intervention sites and by 0.8% (p = 0.003) at control sites (difference -0.5%, p = 0.001). Immediately following implementation, the proportion of childhood notification was +1.5% (p = 0.52) at intervention sites and -4.4% (p = 0.008) at control sites (difference +6.0%, p = 0.08). The proportion of childhood notifications then trended upward by 0.9% (p = 0.23) each quarter at intervention sites relative to the pre-implementation period, while it declined by -1.2% (p = 0.001) at control sites (difference +2.1%, p = 0.042). As shown in **Fig 3,** the maximal effect of the strategy was delayed by many months following initial roll-out, when it was observed that childhood TB notifications accounted for >15% of all TB notifications at the intervention sites in Q4 2020 (20.9%) and Q1 2021 (16.4%); the proportion of quarterly childhood notifications never exceeded 6.4% in the implementation period.

## Discussion

In this study, we demonstrated that implementation of a combination of interventions that target barriers at each step of the childhood TB diagnostic cascade can improve TB case detection at primary health care level. During the study period, the proportion of childhood TB notifications on average increased by almost 1% each quarter at intervention sites compared to the pre-implementation period–resulting in 352 additional notifications, while declining by more than 1% each quarter at control clinics compared to the pre-implementation period–this represented a significant difference of more than 2% per quarter between intervention and control sites. It was especially notable that the proportional contribution of childhood TB notifications exceeded 15% for two consecutive quarters during the study period at intervention sites, after consistently remaining less than 5% prior to implementation. Most of the childhood TB diagnosed (86%) were identified under facility-based activities compared to household contact tracing, however, there was a high case detection yield through both types of activities (10.5% and 6.9%, respectively); the large majority of all childhood TB cases were clinically diagnosed (88%).

The multicomponent childhood TB detection strategy was associated with a nearly threefold increase (508 vs. 156) in notifications despite a shorter follow-up period. Available evidence suggests studies whose strategies focused on strengthening both facility-based case finding and household contact tracing [29–31] resulted into a higher increase in childhood TB notifications than studies that strengthened only contact tracing [32]. This is because, as observed by our study and other studies in both children [29, 33] and adults [34–36], facility-based TB case finding has a higher yield than contact tracing. On the other hand, contact tracing has advantages over facility-based screening as it facilitates earlier TB case detection by overcoming barriers to timely time care-seeking and diagnosis—which limits transmission of TB, is an opportunity to provide health education on TB and TB infection control, and is also a key entry point for TPT. It is thus prudent to adopt a synergistic TB approach that integrates facility and community approaches for optimal childhood case finding and treatment support.

Our findings demonstrate that it is feasible to optimize childhood TB case detection at the primary health care level through a multifaceted approach that addresses key barriers during the diagnostic cascade. This is especially notable because the first point of contact of children with TB with the health system is likely to be the primary healthcare facilities. Strengthening TB case detection at lower levels of care, therefore provides an opportunity for early diagnosis of TB and timely initiation of treatment. It is important to highlight that while we cannot exclude the fact that the nationwide multicomponent strategy implemented by the NTLP, during the study period, to bolster TB detection during the COVID-19 pandemic may have partially contributed to the increased childhood TB case notifications at the study sites [27], this initiative did not have an explicit focus on childhood TB detection and it had no impact on the proportion of childhood TB notifications at national level, both in 2020 and 2021 [1, 37]. Further, at the two control sites, childhood TB notifications actually decreased during this period despite the roll-out of the national TB initiative.

Most of the participants screened (and ultimately TB diagnoses made) under our study were identified through facility-based activities. This may in part be attributable to the awareness and demand generated by the 2 hourly health talks each day, accompanied by an invitation for fast-track TB screening and capacity development among health care workers. The frequency of the health talks increased the reach of messages on childhood TB, which could have addressed some knowledge gaps among community members; several studies, including in Zambia, have demonstrated persistent knowledge gaps and stigma towards childhood TB in the community [38–42]. Indeed, many mothers who presented to the fast-track desk highlighted that they had been alerted to the possibility of their children having TB by the health talks. The fast-track TB services addressed the issue of long waiting time, which is a common barrier to TB services in many settings [43, 44]. Assigning dedicated healthcare workers to provide the health talks and to staff the fast-track desk increased accountability and adherence to the diagnostic algorithms. This, coupled with increased demand for TB screening and the capacity development of health care workers—which increased their index of suspicion of childhood TB–likely resulted in a higher number of children screened for TB.

Most TB cases in our study were clinically diagnosed. This is concordant with findings on childhood TB from other studies [45–47]. Importantly, about 10% of our childhood notifications were bacteriologically confirmed, which is the expected proportion in children [48]. This is worth emphasizing because often times clinicians do not start treatment if they don’t have a positive microbiological result, despite the many known challenges associated with confirming a diagnosis of TB in children [49]. To minimise this, healthcare workers should have capacity built on use of the algorithms to make a clinical diagnosis, while also making the diagnostic algorithms more widely known and available. We also observed that the TB notifications among under children <5 years old were associated with a substantial proportion of the total childhood TB notifications (72%). While this may suggest some degree of overdiagnosis of TB among this age group [48], in our study, it is more likely due to having a significantly smaller number of children ≥5 years being screened for TB relative to those <5 years.

Strengthening access to CXR through provision of an ultraportable CXR machine was a key factor for improving total TB cases detected. As indicated above, most childhood TB cases are clinically diagnosed and a CXR suggestive of TB is one of the main criteria required to make a clinical diagnosis [19]. By making chest x-ray readily available, clinicians are availed more information to either diagnose or exclude childhood TB. Further, the use of urine LAM was also useful in increasing the detection of childhood TB. Currently, its use is only recommended among people living with HIV [25]. However, in our study, we also used it for screening and diagnosis of all children <5 years of age irrespective of HIV status. This is because <5 children have a high risk of disseminated TB [50, 51], one of the pathways through which LAM can be found in urine [52], and is further supported by data suggesting potential utility for LAM testing in HIV negative children <5 years old [23, 24]. However, additional studies are required to determine the sensitivity and specificity of LAM in this patient population.

It was notable that large initial and subsequent declines in childhood TB notifications were observed at control sites during the implementation period. Given the timing during which this occurred, it is most likely attributable to the COVID-19 pandemic which affected global TB notifications [1]. However, while Zambia did experience a large initial decline in total notifications over the first few months of the pandemic, due to the a coordinated multicomponent TB response strategy TB led by the NTLP in conjunction with stakeholders, further negative effects were mitigated and notifications returned to pre-pandemic levels [27]. It is therefore, unclear why childhood notifications at the control sites continued to decline during the study period, and further quality improvement assessments will be performed. Nonetheless, the decreased childhood TB detection during study period at control sites, provides further evidence to support the effectiveness of our multicomponent strategy when taken in conjunction with the positive effects observed at intervention sites.

Our study has some important strengths and limitations. A key strength included the use of routine, programmatic data from two intervention and two control facilities, that provided repeated measures before and during implementation that allowed for evaluation of changes to the level and slope of TB notifications possibly due to the multicomponent strategy and also account potential confounding events and activities. Additional data collected by the study team provided more granularity to the contributions of facility-based activities and contact tracing and the importance of increased CXR screening availability. This study ultimately provides evidence of effectiveness and lessons learnt during implementation of a combination of strategies to optimize TB case detection at primary health facility level.

However, our study provided additional human resource infrastructure to facilitate these strategies, which may mean components like fast-track services, may not be feasible to implement in some resource constrained settings. Additionally, in our study, a positive LAM could be considered bacteriologically confirmed TB at the clinician’s discretion. This may have resulted in a small amount of overdiagnosis of TB as LAM has reduced specificity in this patient population [23–25]. Further, there were slight differences between the total diagnosed TB cases reported using data from the study register and those reported in the district TB notification report. However, these differences were reassuringly very small, and it is likely that this is because on limited occasions, the facility received children in whom a diagnosis of TB had already been made. Additionally, because the intervention and control communities do not have a physical geographical barrier dividing them, we cannot exclude the possibility that parents from the control communities sought evaluation for their children at the intervention sites, which could overestimate the intervention effect size. However, we believe this was unlikely as most individuals living in communities in Lusaka seek care for themselves and their children at public health facilities closest to where they live, and no community-based awareness campaigns were undertaken to promote enhanced pediatric services at the intervention facilities. Finally, given the operational and pragmatic nature of the study, we did not collect data on implementation outcomes such as fidelity to strategy components or perceived feasibility and acceptability among healthcare workers. As these implementation outcomes were not directly assessed, it difficult to know which components were implemented with the highest fidelity and may therefore have most strongly contributed to the positive effects observed in the intervention sites (e.g., what components are the most “essential”), and whether these positive effects could have been even greater in magnitude. Nonetheless, each strategy component likely had an important effect on facilitating increased childhood TB diagnoses and the in general, the most important mechanisms by which multicomponent active case finding strategies result in behavior change and increased TB diagnoses is often difficult to disentangle [53].

In conclusion, implementation of a standardized package of strategies was feasible and was associated with increased childhood TB notifications at the primary health care level in Lusaka, Zambia. Efforts to strengthen childhood TB case detection should combine facility-based case finding and household contact tracing activities coupled with capacitating of healthcare workers, awareness building and demand creation activities, and increased access to improved diagnostic tools.

## Supporting information

S1 Table Childhood TB notifications pre- and post-implementation by site. (DOCX)

S2 Table The proportion of all TB notifications that are accounted for by childhood notifications according to intervention and control sites. (DOCX)

S1 Appendix Manuscript data. (XLS)
