# Supplementary material for: Strategies to increase childhood tuberculosis case detection at the primary health care level: Lessons from an active case finding study in Zambia
Source: PLoS One. 2023 Jul 19;18(7):e0288643. doi: 10.1371/journal.pone.0288643 (PMC10355435; doi:10.1371/journal.pone.0288643)
Supplement: S1 Table — (DOCX) [file pone.0288643.s001.docx]

**S1 Table. Childhood TB notifications pre- and post-implementation by site**

| **Type of patient** | **TB notifications**  **pre-implementation**  **(Jan. 2018- Sept.2019)** | **TB notifications**  **post-implementation**  **(Jan. 2020- Sept. 2021)** | **Difference in notifications (% Change)** |
| --- | --- | --- | --- |
| **Intervention sites** | | | |
| Children <15 years | 156 | 508 | +352 (225.6%) |
| Age <5 years | 61 | 367 | +306 (501.6%) |
| Age 5-14 years | 95 | 141 | +46 (48.4%) |
| Individuals ≥ 15 years | 4069 | 4117 | +48 (1.2%) |
| **Control Sites** | | | |
| Children <15 years | 274 | 189 | -85(-31%) |
| Age <5 years | 169 | 109 | -60 (-35.5%) |
| Age 5-14 years | 105 | 80 | -25 (-23.8%) |
| Individuals ≥ 15 years | 4335 | 3791 | -544 (-12.5%) |
