# Supplementary material for: Strategies to increase childhood tuberculosis case detection at the primary health care level: Lessons from an active case finding study in Zambia
Source: PLoS One. 2023 Jul 19;18(7):e0288643. doi: 10.1371/journal.pone.0288643 (PMC10355435; doi:10.1371/journal.pone.0288643)
Supplement: S2 Table — (DOCX) [file pone.0288643.s002.docx]

|  | **Proportion of TB notifications (%)**  **Before strategy implementation**  **(Jan. 2018 – Dec. 2019)**  **[Period #1]** | |  | **Proportion of TB notifications (%)**  **After strategy implementation**  **(Jan. 2020 – Sept. 2021)**  **[Period #2]** | | |
| --- | --- | --- | --- | --- | --- | --- |
|  | **Initial proportion of quarterly notifications**  **(95%CI)** | **Quarterly trend in childhood TB proportion**  **(95%CI)** |  | **Immediate impact on quarterly childhood TB proportion** | **Quarterly trend in childhood TB proportion** | |
|  |  |  |  | **Number**  **(95%CI)** | **Relative to period #1**  **(95%CI)** | **Overall during period #2**  **(95%CI)** |
| **All children (0-14.9 year of age)** |  |  |  |  |  |  |
| Intervention sites | 2.7%  (2.4, 3.0) | +0.3%^***^  (0.2, 0.4) |  | +1.5%  (-3.5, 6.6) | +0.9%  (-0.7, 2.5) | +1.2%  (-0.4, 2.8) |
| Control sites | 3.5%  (1.7, 5.1) | +0.8%^**^  (0.3, 1.3) |  | -4.4%^**^  (-7.5, -1.4) | -1.2%^**^  (-1.8, -0.6) | -0.4^**^  (-0.6, -0.2) |
| *Difference (relative to control sites)* | -0.8%  (-1.8, 0.2) | -0.5^***^  (-0.8, -0.3) |  | +6.0%^#^  (-0.7, 12.7) | +2.1*  (0.1, 4.2) | +1.6  (-0.4, 3.6) |

**S2 Table. The proportion of all TB notifications that are accounted for by childhood notifications according to intervention and control sites**

^#^p=0.05-0.10, *p<0.05, **p<0.01, ***p<0.001
